# Predictors of half-marathon performance in male recreational athletes

**DOI:** 10.17179/excli2023-6198

**Published:** 2023-06-22

**Authors:** Pantelis T. Nikolaidis, Beat Knechtle

**Affiliations:** 1 School of Health and Caring Sciences, University of West Attica, Egaleo, Greece; 2 Exercise Physiology Laboratory, Nikaia, Greece; 3 Institute of Primary Care, University of Zurich, Zurich, Switzerland; 4 Medbase St. Gallen am Vadianplatz, St. Gallen, Switzerland

**Keywords:** body fat, endurance, ergometry, long-distance running, performance group

## Abstract

Few research has been conducted on predictors of recreational runners' performance, especially in half-marathon running. The purpose of our study was (a) to investigate the relationship of half-marathon race time with training, anthropometry and physiological characteristics, and (b) to develop a formula to predict half-marathon race time in male recreational runners. Recreational runners (n=134, age 44.2±8.7 years; half-marathon race time 104.6±16.2 min) underwent a physical fitness battery consisting of anthropometric and physiological tests. The participants were classified into five performance groups (fast, 73-92 min; above average, 93-99 min; average 100-107 min; below average, 108-117 min; slow group, 118-160 min). A prediction equation was developed in an experimental group (EXP, n=67), validated in a control group (CON, n=67) and prediction bias was estimated with 95 % confidence intervals (CI). Performance groups differed in half-marathon race time, training days, training distance, age, weight, (body mass index) BMI, body fat (BF) and maximum oxygen uptake (VO_2_max) (p≤0.001, η^2^≥0.132), where faster groups had better scores than the slower groups. Half-marathon race time correlated with physiological, anthropometric and training characteristics, with the faster the runner, the better the score in these characteristics (*e.g.*, VO_2_max, r=0.59; BMI, r=-0.55; weekly running distance, r=-0.53, p<0.001). Race time in EXP might be calculated (R^2^=0.63, standard error of the estimate=9.9) using the equation 'Race time (min)=80.056+2.498×BMI-0.594×VO_2_max-0.191×weekly training distance in km'. Validating this formula in CON, no bias was shown (difference between observed and predicted value 2.3±12.8 min, 95 % CI -0.9, 5.4, p=0.153). Half-marathon race time was related to and could be predicted by BMI, VO_2_max and weekly running distance. Based on these relationships, a prediction formula for race time was developed providing a practical tool for recreational runners and professionals working with them.

## Introduction

Half-marathon (HM) has been a running race of increasing popularity with a number of finishers even larger than that of a marathon race (Knechtle et al., 2016[13]; Leyk et al., 2007[14]; Nikolaidis and Knechtle, 2022[21]). For instance, an analysis of about half a million runners competing in HM and marathon races in Switzerland during 1999-2014 showed an increase in HM finishers from 7,767 (1999) to 48,061 (2014) (Knechtle et al., 2016[13]). This trend in participation indicated that a large number of 'novice' runners engaged in this sport recently, and consequently, highlighted the importance to provide scientific support to this population with regards to aspects such as injury prevention (Armento et al., 2023[3]; Martinez-Cano et al., 2021[16]), training characteristics (Fokkema et al., 2020[7]; Špenko et al., 2022[25]), sex and age differences in participation and performance (Nikolaidis et al., 2021[19]; Yang et al., 2022[30]). 'Novice' runners need guidance in setting optimal training goals and for this purpose, several studies attempted to identify predictors of HM race time (Alvero-Cruz et al., 2019[1]; Gómez-Molina et al., 2017[10]; Knechtle et al., 2014[12]).

A comprehensive systematic review of studies on performance predictors in HM showed three broad categories of variables that influenced HM performance: anthropometry, physiological characteristics, and training (Alvero-Cruz et al., 2020[1]). With regards to anthropometry, a faster race time was related with a lower body mass, a lower body mass index (BMI) and a lower body fat percentage (BF) (Alvero-Cruz et al., 2019[2]; Friedrich et al., 2014[8]; Gómez-Molina et al., 2017[10]; Rüst et al., 2011[24]). Physiological characteristics included variables related to aerobic capacity such as performance in Cooper test, velocity at maximal oxygen uptake (VO_2_max) and running velocity at specific lactate concentration (Alvero-Cruz et al., 2019[2]; Muñoz et al., 2012[18]; Roecker et al., 1998[23]). Running experience and running speed in training were among the training characteristics associated with race time (Gómez-Molina et al., 2017[10], Knechtle et al., 2014[12]).

The existing studies on performance predictors in HM have enhanced our knowledge about correlates of performance in this race format; however, a larger set of candidate predictors - than those used previously - should be considered in order to provide a more comprehensive coverage of this topic. Alvero-Cruz and colleagues (2020[1]) reported few studies that examined simultaneously physiological, anthropometric and training data. Therefore, the purpose of the present research was to (a) compare the profile of recreational runners with different HM race time, (b) examine the association of race time with these characteristics, and (c) develop a prediction formula of HM race time in a sub-sample and validate it in another sub-sample. We hypothesized that HM time would relate with physiological, anthropometric, and training characteristics. 

## Methods

### Participants and study design

Participants were male recreational runners (n=134; best HM race time 104.6±16.2 min, number of finished HM races 13.5±18.1, 4.4±1.2 training days per week, training distance 53.1±21.1 km per week, age 44.2±8.7 years, height 176.3±5.8 cm, weight 77.0±9.4 kg, BMI 24.7±2.6 kg.m^-2^ and BF 17.7±4.1 %), who were recruited through public calls using social media. Inclusion criteria were their successful participation in the Athens authentic marathon in 2017 and that they were free of injury or illness (Table 1(Tab. 1)). All data were available in a supplementary file (Training, anthropometric and physiological characteristics of participants).

For the purpose of this cross-sectional study, they underwent a series of physical fitness tests and provided information about performance and training aspects including their best HM race time. The protocols we applied followed the guidelines of the Declaration of Helsinki, and the local Institutional Review Board (EPL 2017/3) approved all procedures. After being informed in detail about the tests and procedures, all participants gave their written consent before the start of the testing session. 

To compare the characteristics of runners by performance level, the participants were grouped according to their best HM race time into quintiles, i.e., fast (73-92 min, n=27), above average (93-99 min, n=27), average (100-107 min, n=27), below average (108-117 min, n=27) and slow group (118-160 min, n=26). In a separate analysis, the participants formed two groups, matched for race time, i.e., an experimental (EXP, n=67) and a control (CON, n=67) group, in order to develop the prediction formula in EXP and validate it in CON.

### Equipment and protocols

A full description of equipment and protocols has been already published in a previous study (Nikolaidis and Knechtle, 2018[20]). Briefly, the participants underwent physiological and anthropometric tests including BMI, BF, VO_2_max, sit-and-reach test (SAR), anaerobic power, squat jump (SJ) and countermovement jump (CMJ). In addition, they provided information about training habits and personal records. All tests were conducted during a single testing session in an exercise physiology laboratory by the same tester.

### Statistical and data analysis

We used IBM SPSS v.23.0 (SPSS, Chicago, IL, USA) for statistical analyses and GraphPad Prism v. 7.05 (GraphPad Software, San Diego, CA, USA) to create figures. The normality of the data was examined by Kolmogorov-Smirnov test and visual inspection of Q-Q plots. The five performance groups were compared using a one-way analysis of variance (ANOVA), Bonferroni post-hoc test and eta squared. Pearson moment correlation coefficient (r) examined the correlations of HM race time with training, anthropometric and physiological characteristics. Differences between EXP and CON (with 95 % confidence intervals, CI), and their magnitude were tested by an independent t-test and Cohen's d, respectively. A prediction formula of HM race time was developed using a stepwise linear regression. Bland-Altman plots were used to examine the agreement between actual and predicted race speed in CON. Alpha was set at 0.05.

## Results

Performance groups differed in race time, training days, training distance, age, weight, BMI, BF and VO_2_max (p≤0.001, η^2^≥0.132), where faster groups had better scores than the slower groups (detailed between group comparisons can be seen in Table 1(Tab. 1)). Except the moderate effect size in age, the magnitude of these differences was large in the abovementioned parameters. No difference was observed among performance groups in the number of finished races, body height, anaerobic power, flexibility and jumping ability (p≥0.059, η^2^≤0.068)

**Figure 1:**
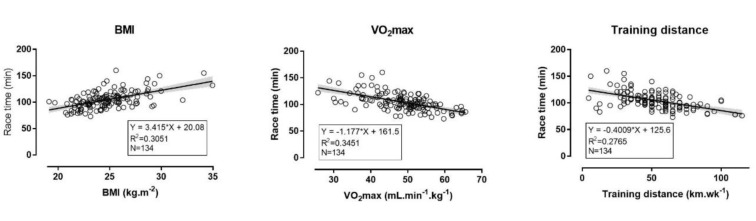
Correlations of half-marathon race time with body mass index, maximal oxygen uptake and weekly running distance in recreational runners (n=134) VO_2_max=maximal oxygen uptake, BMI=body mass index

HM race time was related largely with the number of weekly training days (r=-0.50, p<0.001) and weekly running distance (r=-0.53, p<0.001), moderately with age (r=0.34, p<0.001), and with small magnitude with the number of finished HM (r=-0.21, p=0.019). In addition, race time was related largely with VO_2_max (r=0.59, p<0.001), BMI (r=-0.55, p<0.001), BF (r=-0.53, p<0.001), and moderately with body mass (r=-0.49, p<0.001), but not with anaerobic muscle power (r=0.15, p=0.088), SJ (r=0.15, p=0.093), CMJ (r=0.13, p=0.139) and SAR (r=0.08, p=0.372) (Figure 1(Fig. 1)). 

In comparison with CON, EXP did not differ in age (mean difference 0.7 years; 95 % CI, -2.3, 3.7), height (1.5 cm; 95 % CI -0.5, 3.5), weight (1.8 kg; 95 % CI -1.4, 5.0), BMI (0.1 kg.m^-2^; 95 % CI -0.8, 1.0), BF (0.6 %; 95 % CI -0.8, 2.0), VO_2_max ( 0.1 mL.min^-1^.kg^-1^; 95 % CI -2.7, 2.8), Pmax (-0.3 W.kg^-1^; 95 % CI -0.8, 0.2), SAR (1.6 cm; 95 % CI -1.3, 4.5), SJ (0.2 cm, 95 % CI -1.3, 1.7), CMJ (0 cm; 95 % CI -1.7, 1.6). Moreover, EXP and CON did not differ in race time (-0.7 min; 95 % CI -6.3, 4.9), training days (0; 95 % CI -0.4, 0.4) and training distance per week (-1.8 km; 95 % CI -9.1, 5.5) (Table 2(Tab. 2)).

**Figure 2:**
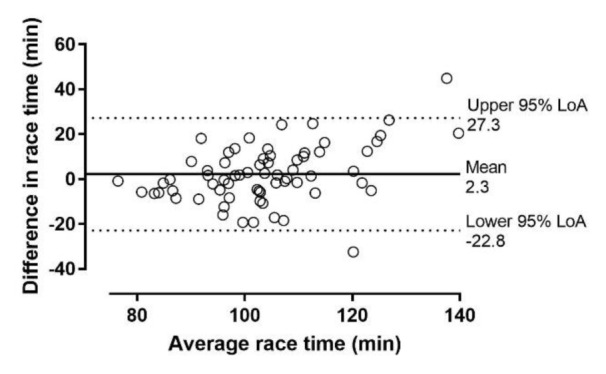
Agreement between actual and predicted half-marathon race time using Bland-Altman plots in recreational runners (n=134). The y axis presents the difference between actual and predicted half-marathon race time, whereas x axis shows the average of actual and predicted score. LoA=limit of agreement

Race time (min) in EXP might be calculated (R^2^=0.63, standard error of the estimate=9.9) using the formula '80.056+2.498×BMI-0.594×VO_2_max-0.191×weekly training distance in km' (Table 3(Tab. 3)). Using this formula in CON, no bias was shown (difference between observed and predicted value 2.3±12.8 min, 95 % CI -0.9, 5.4, p=0.153) (Figure 2(Fig. 2)). 

## Discussion

The main findings of the present study were that (a) male fast HM runners had superior profile with regards to weekly training days and distance, age, weight, BMI, BF and VO_2_max compared to their slower counterparts, (b) HM race time was related with the number of training days, running distance per week, body weight, BMI, BF, VO_2_max and age, and (c) performance in HM could be predicted by a combination of anthropometric (BMI), physiological (VO_2_max) and training characteristics (running distance per week).

The first predictor of HM race time in the stepwise regression - accounting for by ~50 % of the variance - was BMI and this observation was in agreement with the existing literature (Campbell, 1985[6]; Gómez-Molina et al., 2017[10]; Rüst et al., 2011[24]). The importance of BMI for this race distance was already highlighted by Campbell (1985[6]), who showed a higher BMI in male non-finishers than in HM finishers and that BMI moderately correlated with running speed (r=-0.41). In addition, Rüst and colleagues (2011[24]) found a large correlation between BMI and race time (r=0.56) in male finishers in the 'Half Marathon Basel', and suggested that BMI was of higher importance than BF for recreational HM runners. Furthermore, Gomez-Molina and colleagues[10] reported a large correlation of BMI with HM race time (r=0.63-0.64) in two samples of male runners and noted the need to combine training and nutrition for an optimal performance. A comparison of different performance groups indicated that faster HM runners had a lower BMI than their slower peers (Ogueta-Alday et al., 2018[22]). BMI was relevant not only for performance, but also for the risk of injuries (Vadeboncoeur et al., 2012[26]); for instance, an analysis of the 'Lage Landen Marathon Eindhoven' showed an increase risk of running injuries for BMI higher than 26 kg.m^-2^ (van Poppel et al., 2016[27]). It should be emphasized that the average BMI of participants in the present sample as well as in the abovementioned studies was close to the normal-overweight limit. Thus, it was concluded that BMI played an important role in the performance of recreational runners likely due to the fact that an increased weight was an extra load that might cause additional fatigue during a race. 

The second predictor of HM race time was VO_2_max, which came to terms with the existing literature (Alvero-Cruz et al., 2019[2]; Billat et al., 1994[4]; Williams and Nute, 1983[28]). For instance, Alvero-Cruz and colleagues found large correlation of HM race time with VO_2_max and anaerobic threshold in recreational runners (Alvero-Cruz et al., 2019[2]). In an early study, Williams and Nute reported that race time in this distance was related very largely with VO_2_max and anaerobic threshold, recreational runners utilized ~80 % of their VO_2_max during the race (Williams and Nute, 1983[28]). Moreover, the relationship between VO_2_max and performance was observed in a study showing that HM race time was related to maximal aerobic speed in male sub-elite long-distance runners (Billat et al., 1994[4]). In their comparative study, Ogueta-Alday and colleagues reported that faster HM runners had higher VO_2_max than their slower counterparts (Ogueta-Alday et al., 2018[22]). The increased demands of VO_2_max might reflect an increased cardiac output rather changes to arteriovenous oxygen difference (Montero et al., 2015[17]). 

The self-reported running distance per week was the third variable in the model resulting from the stepwise regression analysis. This observation was in agreement with previous studies that showed that the more kilometers the athlete ran, the faster he was in the race (Campbell, 1985[6]; Ogueta-Alday et al., 2018[22]; Rüst et al., 2011[24]). An interpretation of this result might be the direct and indirect impact of regular endurance training on HM race. On one hand, regular endurance training would result in decreasing BMI and increasing VO_2_max, and consequently, in influencing the other two predictors (Li et al., 2021[15]; Williams, 2013[29]). On the other hand, it would result in directly improving performance considering its affinity with the demands of the race (training principle of specificity) (Brooke and Knowles, 1974[5]; Gamble, 2006[9]; Kasper, 2019[11]).

A limitation of the present study was that performance in HM race was defined as the best personal record in this distance, which was self-reported by participants. Although there was no reason to assume deviation in the self-reported from the actual score, a concern might be related to the time period between the occurrence of the best personal performance and the date of the testing session. Nevertheless, strength of the study was that it considered a larger number of candidate predictors than previous studies providing a more comprehensive coverage of this topic. For instance, previous research (Campbell, 1985[6]; Rüst et al., 2011[24]) did not include physiological data in the development of prediction equation for HM performance. 

## Conclusions

In summary, the present study developed a prediction equation of HM race time that should be further tested before its use in practice by male recreational runners. Furthermore, the findings highlight the need to optimize body weight through optimal exercise and nutritional interventions.

## Declaration

### Author contribution statement

Pantelis T. Nikolaidis collected the data and drafted the manuscript and Beat Knechtle helped in drafting and editing the manuscript.

### Funding

No funding.

### Conflict of interest

No conflict of interest.

### Data availability

All data are available by the corresponding author upon reasonable request.

## Supplementary Material

Supplementary information

## Figures and Tables

**Table 1:**
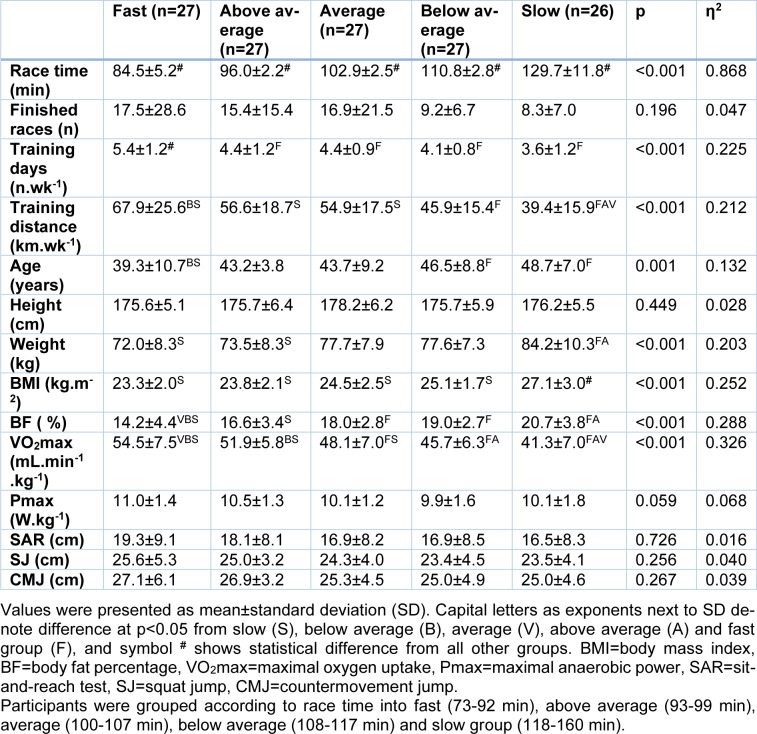
Performance characteristics of participants (n=134) and by race time

**Table 2:**
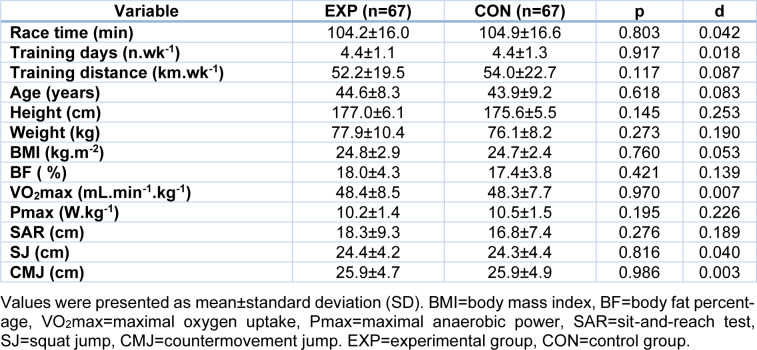
Performance characteristics of participants (experimental *versus* control group)

**Table 3:**
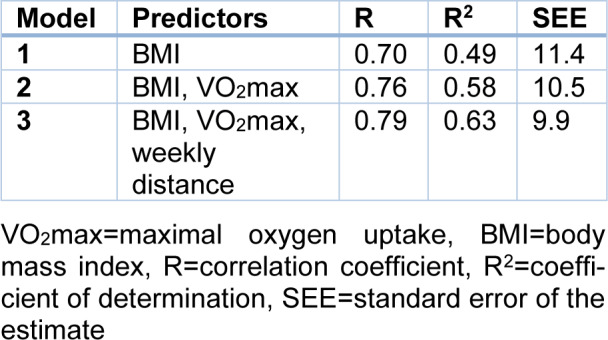
Model summary of stepwise regression in the experimental group (n=67)
